# 12β,14-Dihy­droxy-3-oxo-5β,20(22)-cardenolide monohydrate

**DOI:** 10.1107/S1600536810038031

**Published:** 2010-10-02

**Authors:** Chao-Jun He, Min Wang, Hua Sun, Zeng-bing Liu, Yu-wan Fu

**Affiliations:** a College of Biotechnology, Tianjin University of Science and Technology, Tianjin 300457, People’s Republic of China

## Abstract

The title compound, digoxigenone, C_23_H_30_O_5_·H_2_O, was biotransformed from digoxigenin. In the crystal, inter­molecular O—H⋯O hydrogen bonds contribute to the formation of a three-dimensional supra­molecular structure. The title compound has three fused six-membered rings (*A*,*B*,*C*) and two non-fused five-membered rings (*D*,*E*). As in other structures, compound nucleus has a *cis*-*trans*-*cis* conformation for the *A*-*B*,*B*-*C*,*C*-*D* ring junctions with  rings *A*, *B* and *C* exhibiting chair conformations.

## Related literature

Digitoxin and digoxin, the typical clinically used forms (Kreis *et al.*, 1998 ▶), are the drugs of choice for the treatment of congestive heart failure, acting as selective inhibitors of the Na+, K+ ATPase enzyme. For the biotransformation of digitoxigenin into digoxigenin and digoxigenone by *Fusarium ciliatum* and into 1 β-hy­droxy­digitoxigenin, 7-β-hy­droxy­digi­toxi­genin, 8-β-hydroxidigitoxigenin and digitoxigenone by *Cochliobolus lunatus*, see: Pádua *et al.* (2005 ▶, 2007 ▶).
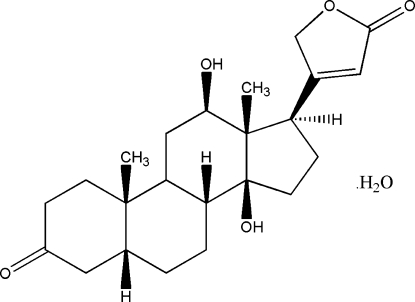


## Experimental

### 

#### Crystal data

C_23_H_30_O_5_·H_2_O
*M* *_r_* = 404.49
Triclinic, 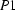

*a* = 7.4017 (15) Å
*b* = 7.7450 (15) Å
*c* = 10.215 (2) Å
α = 99.51 (3)°
β = 94.70 (3)°
γ = 114.97 (3)°
*V* = 516.0 (2) Å^3^
*Z* = 1
Mo *K*α radiation
μ = 0.09 mm^−1^
*T* = 293 K
0.32 × 0.26 × 0.20 mm

#### Data collection

Rigaku Saturn CCD area-detector diffractometer
Absorption correction: multi-scan (*CrystalClear*; Rigaku, 2005 ▶) *T*_min_ = 0.971, *T*_max_ = 0.982
5251 measured reflections
1809 independent reflections
1443 reflections with *I* > 2σ(*I*)
*R*_int_ = 0.035

#### Refinement

*R*[*F* ^2^ > 2σ(*F* ^2^)] = 0.034
*wR*(*F* ^2^) = 0.082
*S* = 1.00
1809 reflections
275 parameters
6 restraints
H atoms treated by a mixture of independent and constrained refinement
Δρ_max_ = 0.15 e Å^−3^
Δρ_min_ = −0.14 e Å^−3^

### 

Data collection: *CrystalClear* (Rigaku, 2005 ▶); cell refinement: *CrystalClear*; data reduction: *CrystalClear*; program(s) used to solve structure: *SHELXS97* (Sheldrick, 2008 ▶); program(s) used to refine structure: *SHELXL97* (Sheldrick, 2008 ▶); molecular graphics: *SHELXTL* (Sheldrick, 2008 ▶); software used to prepare material for publication: *SHELXTL*.

## Supplementary Material

Crystal structure: contains datablocks global, I. DOI: 10.1107/S1600536810038031/hg2696sup1.cif

Structure factors: contains datablocks I. DOI: 10.1107/S1600536810038031/hg2696Isup2.hkl

Additional supplementary materials:  crystallographic information; 3D view; checkCIF report

## Figures and Tables

**Table 1:** Hydrogen-bond geometry (Å, °)

*D*—H⋯*A*	*D*—H	H⋯*A*	*D*⋯*A*	*D*—H⋯*A*
O2—H2⋯O6^i^	0.82	1.99	2.808 (3)	173
O3—H3⋯O2^ii^	0.82	2.10	2.900 (3)	164
O6—H1*W*⋯O1^iii^	0.86 (1)	1.94 (1)	2.801 (3)	174 (4)
O6—H2*W*⋯O5^iv^	0.86 (1)	1.91 (2)	2.741 (4)	162 (5)

Symmetry codes: (i) 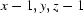; (ii) 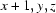; (iii) 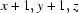; (iv) 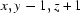.

**Fig. 1.**
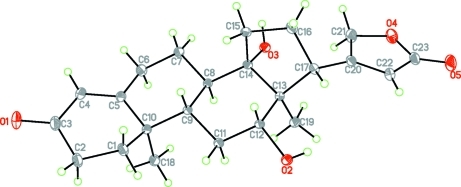
Perspective view of the title compound (noncoordinated water molecule was omitted for clarity)

**Fig. 2.**
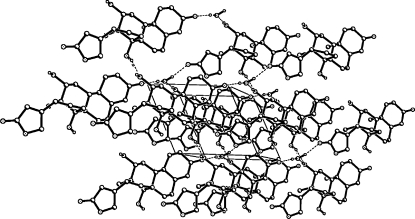
View of the packing of the title compound

## supplementary crystallographic information

### Comment

Digitoxin and digoxin, the typical clinically used forms (Kreis *et al.*, 1998), are the drugs of choice for the treatment of congestive heart failure, acting as selective inhibitors of the Na+, K+ ATPase enzyme. Digitoxigenin has been biotransformed into digoxigenin and digoxigenone by Fusarium ciliatum and into 1 beta-hydroxydigitoxigenin, 7 beta-hydroxydigitoxigenin, 8 beta-hydroxidigitoxigenin, and digitoxigenone by Cochliobolus lunatus (Pádua *et al.*, 2005; Pádua *et al.*, 2007). In this paper, we studied the biotransformation of digoxigenin by Arthrobacter simplex and reported crystal structure of the compound digoxigenone.

The crystal structure is shown in Fig. 1. The crystal structure consists of an digoxigenone molecule and one water molecule. Compound digoxigenone has three fused six-membered rings (A/B/C) and two non-fused five-membered rings (D/E). As in other structures the A, B and C rings have a chair conformation. The 12-hydroxy is beta configuration with the torsion angles C9—C11—C12—O2 = -179.6 (2)°. The 14-hydroxy is beta configuration with the torsion angles C7—C8—C14—O3 = -64.4 (3)°. The orientation of the lactone ring is determined by the torsion angle C(13)—C(17)—C(20)—C(22) = -113.1 (3)°.

In the crystal packing (Fig. 2), X-ray analysis indicates that there were three types of intermolecular hydrogen bonds contributed to the formation of three-dimensional supramolecular structure: between solvent water molecule and carbonyl of adjacent digoxigenone molecule, hydroxyl of digoxigenone molecule and hydroxyl of adjacent digoxigenone molecule, and hydroxyl of digoxigenone molecule and solvent water molecule. The hydrogen bonding parameters are O(6)—H(6 A)···O(1)#3, 2.800 (3), 173.7; O(6)—H(6B)···O(5)#4, 2.742 (3), 160.3; O(2)—H(2)···O(6)#1 2.805 (2), 172.7; and O(3)—H(3)···O(2)#2, 2.901 (2), 163.7. (Symmetry code: #1 *x* - 1,*y*,*z* - 1; #2 *x* + 1,*y*,*z*; #3 *x* + 1,*y* + 1,*z*; #4 *x*,*y* - 1,*z* + 1).

### Experimental

The Arthrobacter simplex TCCC 11037 (stored in our laboratory) was maintained at 277 K on a slant containing glucose 10.0 g/*L*, yeast extract 10.0 g *L*-1 and agar 20.0 g *L*-1 (pH = 7.2). Seed media consists of glucose 10.0 g/*L*, corn slurry 10.0 g/*L*, peptone 5.0 g/*L* and KH_2_PO_4_ 2.5 g/*L* (pH = 7.2).

The Arthrobacter simplex (ASP) cells were prepared in two consecutive cultivation steps (18 h for seed culture and 24 h for cell incubation, respectively) in shake flasks. The whole ASP culture using 5% (*v*/*v*) of seed culture was grown in 250 ml shake flasks containing 30 ml culture media on a rotary shaker (160 rpm) at 305 K. Digoxigenin dissolved in ethanol (50 g *L*-1) and distributed among the flasks (0.34 g/*L*),and then the reaction was allowed to proceed for 7 days at 307 K. The biotransformation products were sequentially extracted with ethyl acetate (3 × 100 ml) in a separator funnel and the solvent was vacuum removed at 333 K, until a residue was produced. The crude extracts were purified by Si gel column using chloroform/methanol (25:1, *v*/*v*). The white power was diffused with petroleum ether/acetone (5:3, *v*/*v*) at room temperature. Colourless prism crystals were obtained.

### Refinement

The C-bound H atoms were geometrically placed (C–H = 0.96Å for methyl, 0.97 Å for methylene and 0.93Å for C(sp2), respectively) and refined as riding with *U*_iso_(H) = 1.5*U*_eq_(C) for methyl and *U*_iso_(H) = 1.2*U*_eq_(C) for others. The H atoms of hyadroxyl were geometrically placed O–H = 0.82Å and refined as riding with *U*_iso_(H) = 1.5*U*_eq_(O). The water H atoms were located from a difference map and refined freely.

### Crystal data

**Table:**

C_23_H_30_O_5_·H_2_O	*Z* = 1
*M**_r_* = 404.49	*F*(000) = 218
Triclinic, *P*1	*D*_x_ = 1.302 Mg m^−^^3^
Hall symbol: P 1	Mo *K*α radiation, λ = 0.71073 Å
*a* = 7.4017 (15) Å	Cell parameters from 1642 reflections
*b* = 7.7450 (15) Å	θ = 2.1–27.8°
*c* = 10.215 (2) Å	µ = 0.09 mm^−^^1^
α = 99.51 (3)°	*T* = 293 K
β = 94.70 (3)°	Prism, colourless
γ = 114.97 (3)°	0.32 × 0.26 × 0.20 mm
*V* = 516.0 (2) Å^3^	

### Data collection

**Table:**

Rigaku Saturn CCD area-detector diffractometer	1809 independent reflections
Radiation source: rotating anode	1443 reflections with *I* > 2σ(*I*)
confocal	*R*_int_ = 0.035
Detector resolution: 7.31 pixels mm^-1^	θ_max_ = 25.0°, θ_min_ = 2.1°
ω and φ scans	*h* = −8→8
Absorption correction: multi-scan (*CrystalClear*; Rigaku, 2005)	*k* = −9→8
*T*_min_ = 0.971, *T*_max_ = 0.982	*l* = −12→12
5251 measured reflections	

### Refinement

**Table:**

Refinement on *F*^2^	Secondary atom site location: difference Fourier map
Least-squares matrix: full	Hydrogen site location: inferred from neighbouring sites
*R*[*F*^2^ > 2σ(*F*^2^)] = 0.034	H atoms treated by a mixture of independent and constrained refinement
*wR*(*F*^2^) = 0.082	*w* = 1/[σ^2^(*F*_o_^2^) + (0.0504*P*)^2^ + 0.010*P*] where *P* = (*F*_o_^2^ + 2*F*_c_^2^)/3
*S* = 1.00	(Δ/σ)_max_ < 0.001
1809 reflections	Δρ_max_ = 0.14 e Å^−^^3^
275 parameters	Δρ_min_ = −0.14 e Å^−^^3^
6 restraints	Extinction correction: *SHELXL97* (Sheldrick, 2008), Fc^*^=kFc[1+0.001xFc^2^λ^3^/sin(2θ)]^-1/4^
Primary atom site location: structure-invariant direct methods	Extinction coefficient: 0.43 (3)

### Special details

**Table:**

Geometry. All e.s.d.'s (except the e.s.d. in the dihedral angle between two l.s. planes) are estimated using the full covariance matrix. The cell e.s.d.'s are taken into account individually in the estimation of e.s.d.'s in distances, angles and torsion angles; correlations between e.s.d.'s in cell parameters are only used when they are defined by crystal symmetry. An approximate (isotropic) treatment of cell e.s.d.'s is used for estimating e.s.d.'s involving l.s. planes.
Refinement. Refinement of *F*^2^ against ALL reflections. The weighted *R*-factor *wR* and goodness of fit *S* are based on *F*^2^, conventional *R*-factors *R* are based on *F*, with *F* set to zero for negative *F*^2^. The threshold expression of *F*^2^ > σ(*F*^2^) is used only for calculating *R*-factors(gt) *etc*. and is not relevant to the choice of reflections for refinement. *R*-factors based on *F*^2^ are statistically about twice as large as those based on *F*, and *R*- factors based on ALL data will be even larger.

### Fractional atomic coordinates and isotropic or equivalent isotropic displacement parameters (Å^2^)

**Table:**

	*x*	*y*	*z*	*U*_iso_*/*U*_eq_	
C1	−0.0073 (4)	0.3043 (5)	0.5686 (3)	0.0474 (8)	
H1A	−0.1000	0.3610	0.5551	0.057*	
H1B	−0.0281	0.2103	0.4864	0.057*	
C2	−0.0581 (5)	0.1979 (5)	0.6826 (3)	0.0566 (9)	
H2A	−0.0574	0.2868	0.7618	0.068*	
H2B	−0.1931	0.0907	0.6569	0.068*	
C3	0.0884 (5)	0.1203 (5)	0.7161 (3)	0.0562 (9)	
C4	0.2931 (5)	0.2275 (5)	0.6930 (3)	0.0548 (8)	
H4	0.3868	0.1813	0.7137	0.066*	
C5	0.3553 (4)	0.3895 (4)	0.6435 (3)	0.0394 (7)	
C6	0.5751 (4)	0.5096 (5)	0.6423 (3)	0.0510 (8)	
H6A	0.6496	0.4382	0.6646	0.061*	
H6B	0.6252	0.6295	0.7107	0.061*	
C7	0.6133 (4)	0.5598 (5)	0.5056 (3)	0.0426 (7)	
H7A	0.5823	0.4415	0.4395	0.051*	
H7B	0.7552	0.6476	0.5118	0.051*	
C8	0.4846 (4)	0.6561 (4)	0.4589 (3)	0.0302 (6)	
H8	0.5236	0.7780	0.5253	0.036*	
C9	0.2593 (3)	0.5255 (4)	0.4572 (3)	0.0299 (6)	
H9	0.2219	0.4043	0.3906	0.036*	
C10	0.2105 (4)	0.4666 (3)	0.5934 (2)	0.0321 (6)	
C11	0.1312 (4)	0.6216 (4)	0.4064 (3)	0.0380 (7)	
H11A	−0.0108	0.5345	0.4005	0.046*	
H11B	0.1622	0.7411	0.4712	0.046*	
C12	0.1679 (4)	0.6685 (4)	0.2704 (3)	0.0333 (6)	
H12	0.1254	0.5461	0.2038	0.040*	
C13	0.3939 (4)	0.8041 (3)	0.2682 (2)	0.0302 (6)	
C14	0.5248 (4)	0.7098 (4)	0.3221 (3)	0.0311 (6)	
C15	0.4892 (4)	0.5418 (4)	0.2060 (3)	0.0364 (6)	
H15A	0.3658	0.4274	0.2068	0.044*	
H15B	0.6013	0.5080	0.2116	0.044*	
C16	0.4720 (4)	0.6179 (4)	0.0779 (3)	0.0441 (7)	
H16A	0.3672	0.5153	0.0083	0.053*	
H16B	0.5991	0.6614	0.0442	0.053*	
C17	0.4186 (4)	0.7903 (4)	0.1164 (3)	0.0350 (6)	
H17	0.2852	0.7522	0.0644	0.042*	
C18	0.2350 (5)	0.6415 (5)	0.7033 (3)	0.0507 (8)	
H18A	0.2182	0.6027	0.7878	0.076*	
H18B	0.1346	0.6836	0.6789	0.076*	
H18C	0.3674	0.7469	0.7116	0.076*	
C19	0.4521 (4)	1.0101 (4)	0.3480 (3)	0.0418 (7)	
H19A	0.5941	1.0896	0.3529	0.063*	
H19B	0.4220	1.0049	0.4373	0.063*	
H19C	0.3767	1.0654	0.3039	0.063*	
C20	0.5633 (4)	0.9736 (4)	0.0803 (3)	0.0380 (6)	
C21	0.7889 (4)	1.0689 (4)	0.1180 (4)	0.0567 (9)	
H21A	0.8296	1.1136	0.2150	0.068*	
H21B	0.8434	0.9787	0.0863	0.068*	
C22	0.5139 (5)	1.0757 (4)	0.0035 (3)	0.0488 (8)	
H22	0.3828	1.0478	−0.0331	0.059*	
C23	0.6953 (5)	1.2364 (4)	−0.0143 (3)	0.0523 (8)	
O1	0.0400 (5)	−0.0230 (4)	0.7666 (3)	0.0905 (10)	
O2	0.0392 (3)	0.7578 (3)	0.2377 (2)	0.0497 (6)	
H2	0.0251	0.7534	0.1566	0.075*	
O3	0.7324 (2)	0.8562 (2)	0.33978 (19)	0.0392 (5)	
H3	0.8017	0.8051	0.3095	0.059*	
O4	0.8586 (3)	1.2327 (3)	0.0526 (2)	0.0610 (6)	
O5	0.7166 (4)	1.3617 (4)	−0.0773 (3)	0.0797 (8)	
O6	0.9639 (4)	0.7538 (4)	0.9630 (3)	0.0661 (7)	
H1W	0.979 (7)	0.816 (5)	0.899 (3)	0.105 (15)*	
H2W	0.901 (7)	0.6285 (16)	0.937 (4)	0.13 (2)*	

### Atomic displacement parameters (Å^2^)

**Table:**

	*U*^11^	*U*^22^	*U*^33^	*U*^12^	*U*^13^	*U*^23^
C1	0.0383 (16)	0.0560 (19)	0.0500 (19)	0.0171 (14)	0.0123 (14)	0.0255 (16)
C2	0.0578 (19)	0.053 (2)	0.061 (2)	0.0180 (16)	0.0204 (16)	0.0302 (16)
C3	0.076 (2)	0.0477 (19)	0.049 (2)	0.0266 (17)	0.0187 (17)	0.0210 (16)
C4	0.070 (2)	0.063 (2)	0.056 (2)	0.0440 (18)	0.0204 (16)	0.0306 (17)
C5	0.0454 (16)	0.0485 (17)	0.0328 (16)	0.0267 (14)	0.0062 (12)	0.0149 (13)
C6	0.0421 (17)	0.075 (2)	0.0494 (19)	0.0327 (16)	0.0062 (14)	0.0296 (17)
C7	0.0332 (16)	0.0563 (18)	0.0503 (18)	0.0265 (14)	0.0103 (13)	0.0232 (15)
C8	0.0299 (13)	0.0342 (14)	0.0291 (14)	0.0165 (11)	0.0042 (10)	0.0076 (11)
C9	0.0297 (13)	0.0332 (14)	0.0311 (14)	0.0168 (11)	0.0066 (10)	0.0099 (11)
C10	0.0342 (15)	0.0356 (16)	0.0300 (15)	0.0179 (12)	0.0057 (11)	0.0089 (12)
C11	0.0336 (15)	0.0534 (17)	0.0390 (16)	0.0261 (13)	0.0104 (12)	0.0205 (14)
C12	0.0263 (13)	0.0449 (16)	0.0383 (16)	0.0213 (12)	0.0098 (11)	0.0166 (13)
C13	0.0309 (13)	0.0318 (14)	0.0306 (15)	0.0156 (11)	0.0074 (11)	0.0083 (11)
C14	0.0234 (13)	0.0334 (14)	0.0380 (15)	0.0127 (11)	0.0087 (11)	0.0096 (12)
C15	0.0371 (14)	0.0359 (15)	0.0399 (16)	0.0179 (12)	0.0138 (12)	0.0093 (13)
C16	0.0528 (17)	0.0400 (15)	0.0382 (17)	0.0183 (13)	0.0133 (13)	0.0083 (13)
C17	0.0322 (13)	0.0384 (15)	0.0311 (14)	0.0117 (12)	0.0056 (11)	0.0096 (12)
C18	0.075 (2)	0.0529 (19)	0.0376 (17)	0.0377 (18)	0.0189 (15)	0.0126 (15)
C19	0.0480 (16)	0.0357 (15)	0.0439 (16)	0.0210 (13)	0.0088 (13)	0.0076 (13)
C20	0.0429 (15)	0.0383 (15)	0.0328 (15)	0.0169 (12)	0.0113 (12)	0.0088 (12)
C21	0.0425 (18)	0.049 (2)	0.074 (2)	0.0101 (15)	0.0141 (15)	0.0295 (17)
C22	0.055 (2)	0.0499 (18)	0.0475 (18)	0.0235 (16)	0.0153 (14)	0.0210 (15)
C23	0.071 (2)	0.0427 (18)	0.0470 (19)	0.0238 (16)	0.0232 (17)	0.0179 (15)
O1	0.119 (2)	0.0708 (17)	0.107 (2)	0.0453 (16)	0.0475 (19)	0.0631 (18)
O2	0.0421 (11)	0.0820 (15)	0.0485 (12)	0.0417 (11)	0.0136 (10)	0.0331 (12)
O3	0.0267 (9)	0.0401 (11)	0.0502 (12)	0.0131 (8)	0.0086 (8)	0.0119 (9)
O4	0.0545 (14)	0.0495 (13)	0.0670 (16)	0.0068 (10)	0.0171 (11)	0.0236 (11)
O5	0.108 (2)	0.0596 (15)	0.083 (2)	0.0347 (15)	0.0367 (15)	0.0438 (14)
O6	0.0627 (15)	0.0775 (19)	0.0564 (15)	0.0232 (15)	0.0116 (12)	0.0316 (14)

### Geometric parameters (Å, °)

**Table:**

C1—C2	1.518 (4)	C13—C19	1.526 (4)
C1—C10	1.536 (4)	C13—C14	1.555 (3)
C1—H1A	0.9700	C13—C17	1.567 (3)
C1—H1B	0.9700	C14—O3	1.448 (3)
C2—C3	1.487 (4)	C14—C15	1.523 (4)
C2—H2A	0.9700	C15—C16	1.538 (4)
C2—H2B	0.9700	C15—H15A	0.9700
C3—O1	1.229 (4)	C15—H15B	0.9700
C3—C4	1.450 (5)	C16—C17	1.547 (4)
C4—C5	1.342 (4)	C16—H16A	0.9700
C4—H4	0.9300	C16—H16B	0.9700
C5—C6	1.497 (4)	C17—C20	1.501 (4)
C5—C10	1.523 (3)	C17—H17	0.9800
C6—C7	1.527 (4)	C18—H18A	0.9600
C6—H6A	0.9700	C18—H18B	0.9600
C6—H6B	0.9700	C18—H18C	0.9600
C7—C8	1.528 (4)	C19—H19A	0.9600
C7—H7A	0.9700	C19—H19B	0.9600
C7—H7B	0.9700	C19—H19C	0.9600
C8—C14	1.539 (3)	C20—C22	1.333 (4)
C8—C9	1.540 (3)	C20—C21	1.497 (4)
C8—H8	0.9800	C21—O4	1.451 (3)
C9—C11	1.537 (3)	C21—H21A	0.9700
C9—C10	1.558 (3)	C21—H21B	0.9700
C9—H9	0.9800	C22—C23	1.445 (4)
C10—C18	1.544 (4)	C22—H22	0.9300
C11—C12	1.510 (4)	C23—O5	1.215 (4)
C11—H11A	0.9700	C23—O4	1.352 (4)
C11—H11B	0.9700	O2—H2	0.8200
C12—O2	1.442 (3)	O3—H3	0.8200
C12—C13	1.560 (3)	O6—H1W	0.861 (11)
C12—H12	0.9800	O6—H2W	0.862 (11)
			
C2—C1—C10	113.8 (2)	C13—C12—H12	108.7
C2—C1—H1A	108.8	C19—C13—C14	113.6 (2)
C10—C1—H1A	108.8	C19—C13—C12	110.0 (2)
C2—C1—H1B	108.8	C14—C13—C12	108.13 (19)
C10—C1—H1B	108.8	C19—C13—C17	114.5 (2)
H1A—C1—H1B	107.7	C14—C13—C17	103.33 (19)
C3—C2—C1	111.8 (3)	C12—C13—C17	106.8 (2)
C3—C2—H2A	109.3	O3—C14—C15	108.79 (19)
C1—C2—H2A	109.3	O3—C14—C8	107.92 (19)
C3—C2—H2B	109.3	C15—C14—C8	116.0 (2)
C1—C2—H2B	109.3	O3—C14—C13	105.59 (19)
H2A—C2—H2B	107.9	C15—C14—C13	104.0 (2)
O1—C3—C4	121.6 (3)	C8—C14—C13	113.95 (19)
O1—C3—C2	121.4 (3)	C14—C15—C16	105.1 (2)
C4—C3—C2	116.9 (3)	C14—C15—H15A	110.7
C5—C4—C3	123.9 (3)	C16—C15—H15A	110.7
C5—C4—H4	118.1	C14—C15—H15B	110.7
C3—C4—H4	118.1	C16—C15—H15B	110.7
C4—C5—C6	120.8 (3)	H15A—C15—H15B	108.8
C4—C5—C10	122.4 (3)	C15—C16—C17	107.2 (2)
C6—C5—C10	116.7 (2)	C15—C16—H16A	110.3
C5—C6—C7	112.2 (2)	C17—C16—H16A	110.3
C5—C6—H6A	109.2	C15—C16—H16B	110.3
C7—C6—H6A	109.2	C17—C16—H16B	110.3
C5—C6—H6B	109.2	H16A—C16—H16B	108.5
C7—C6—H6B	109.2	C20—C17—C16	112.9 (2)
H6A—C6—H6B	107.9	C20—C17—C13	116.6 (2)
C6—C7—C8	111.6 (2)	C16—C17—C13	105.62 (19)
C6—C7—H7A	109.3	C20—C17—H17	107.1
C8—C7—H7A	109.3	C16—C17—H17	107.1
C6—C7—H7B	109.3	C13—C17—H17	107.1
C8—C7—H7B	109.3	C10—C18—H18A	109.5
H7A—C7—H7B	108.0	C10—C18—H18B	109.5
C7—C8—C14	111.8 (2)	H18A—C18—H18B	109.5
C7—C8—C9	110.3 (2)	C10—C18—H18C	109.5
C14—C8—C9	112.24 (18)	H18A—C18—H18C	109.5
C7—C8—H8	107.4	H18B—C18—H18C	109.5
C14—C8—H8	107.4	C13—C19—H19A	109.5
C9—C8—H8	107.4	C13—C19—H19B	109.5
C11—C9—C8	109.72 (19)	H19A—C19—H19B	109.5
C11—C9—C10	112.22 (19)	C13—C19—H19C	109.5
C8—C9—C10	114.26 (19)	H19A—C19—H19C	109.5
C11—C9—H9	106.7	H19B—C19—H19C	109.5
C8—C9—H9	106.7	C22—C20—C21	107.7 (2)
C10—C9—H9	106.7	C22—C20—C17	125.9 (3)
C5—C10—C1	109.3 (2)	C21—C20—C17	126.4 (2)
C5—C10—C18	107.8 (2)	O4—C21—C20	105.0 (2)
C1—C10—C18	110.2 (2)	O4—C21—H21A	110.7
C5—C10—C9	109.2 (2)	C20—C21—H21A	110.7
C1—C10—C9	108.2 (2)	O4—C21—H21B	110.7
C18—C10—C9	112.1 (2)	C20—C21—H21B	110.7
C12—C11—C9	112.8 (2)	H21A—C21—H21B	108.8
C12—C11—H11A	109.0	C20—C22—C23	109.6 (3)
C9—C11—H11A	109.0	C20—C22—H22	125.2
C12—C11—H11B	109.0	C23—C22—H22	125.2
C9—C11—H11B	109.0	O5—C23—O4	120.3 (3)
H11A—C11—H11B	107.8	O5—C23—C22	130.4 (3)
O2—C12—C11	106.0 (2)	O4—C23—C22	109.3 (2)
O2—C12—C13	111.4 (2)	C12—O2—H2	109.5
C11—C12—C13	113.1 (2)	C14—O3—H3	109.5
O2—C12—H12	108.7	C23—O4—C21	108.4 (2)
C11—C12—H12	108.7	H1W—O6—H2W	114.3 (19)
			
C10—C1—C2—C3	−54.0 (4)	C7—C8—C14—O3	−64.4 (3)
C1—C2—C3—O1	−153.4 (3)	C9—C8—C14—O3	171.01 (18)
C1—C2—C3—C4	29.7 (4)	C7—C8—C14—C15	57.9 (3)
O1—C3—C4—C5	−177.1 (3)	C9—C8—C14—C15	−66.7 (3)
C2—C3—C4—C5	−0.2 (5)	C7—C8—C14—C13	178.7 (2)
C3—C4—C5—C6	170.4 (3)	C9—C8—C14—C13	54.1 (3)
C3—C4—C5—C10	−6.1 (5)	C19—C13—C14—O3	−48.0 (3)
C4—C5—C6—C7	131.9 (3)	C12—C13—C14—O3	−170.35 (19)
C10—C5—C6—C7	−51.3 (4)	C17—C13—C14—O3	76.7 (2)
C5—C6—C7—C8	53.6 (4)	C19—C13—C14—C15	−162.5 (2)
C6—C7—C8—C14	179.2 (2)	C12—C13—C14—C15	75.2 (2)
C6—C7—C8—C9	−55.2 (3)	C17—C13—C14—C15	−37.8 (2)
C7—C8—C9—C11	−178.5 (3)	C19—C13—C14—C8	70.3 (3)
C14—C8—C9—C11	−53.1 (3)	C12—C13—C14—C8	−52.1 (3)
C7—C8—C9—C10	54.5 (3)	C17—C13—C14—C8	−165.0 (2)
C14—C8—C9—C10	179.9 (2)	O3—C14—C15—C16	−75.9 (2)
C4—C5—C10—C1	−17.3 (4)	C8—C14—C15—C16	162.2 (2)
C6—C5—C10—C1	166.1 (3)	C13—C14—C15—C16	36.3 (2)
C4—C5—C10—C18	102.6 (3)	C14—C15—C16—C17	−20.6 (3)
C6—C5—C10—C18	−74.1 (3)	C15—C16—C17—C20	125.5 (2)
C4—C5—C10—C9	−135.4 (3)	C15—C16—C17—C13	−3.0 (3)
C6—C5—C10—C9	47.9 (3)	C19—C13—C17—C20	22.6 (3)
C2—C1—C10—C5	46.7 (3)	C14—C13—C17—C20	−101.5 (2)
C2—C1—C10—C18	−71.5 (3)	C12—C13—C17—C20	144.6 (2)
C2—C1—C10—C9	165.6 (2)	C19—C13—C17—C16	148.9 (2)
C11—C9—C10—C5	−175.0 (2)	C14—C13—C17—C16	24.8 (2)
C8—C9—C10—C5	−49.3 (3)	C12—C13—C17—C16	−89.1 (2)
C11—C9—C10—C1	66.1 (3)	C16—C17—C20—C22	124.3 (3)
C8—C9—C10—C1	−168.16 (19)	C13—C17—C20—C22	−113.1 (3)
C11—C9—C10—C18	−55.6 (3)	C16—C17—C20—C21	−52.4 (4)
C8—C9—C10—C18	70.1 (3)	C13—C17—C20—C21	70.1 (4)
C8—C9—C11—C12	55.3 (3)	C22—C20—C21—O4	−0.7 (3)
C10—C9—C11—C12	−176.6 (2)	C17—C20—C21—O4	176.6 (2)
C9—C11—C12—O2	−179.6 (2)	C21—C20—C22—C23	0.4 (3)
C9—C11—C12—C13	−57.3 (3)	C17—C20—C22—C23	−176.9 (2)
O2—C12—C13—C19	48.2 (3)	C20—C22—C23—O5	179.4 (3)
C11—C12—C13—C19	−71.1 (3)	C20—C22—C23—O4	0.1 (3)
O2—C12—C13—C14	172.8 (2)	O5—C23—O4—C21	−179.9 (3)
C11—C12—C13—C14	53.5 (3)	C22—C23—O4—C21	−0.5 (3)
O2—C12—C13—C17	−76.6 (2)	C20—C21—O4—C23	0.7 (3)
C11—C12—C13—C17	164.1 (2)		

### Hydrogen-bond geometry (Å, °)

**Table:**

*D*—H···*A*	*D*—H	H···*A*	*D*···*A*	*D*—H···*A*
O2—H2···O6^i^	0.82	1.99	2.808 (3)	173
O3—H3···O2^ii^	0.82	2.10	2.900 (3)	164
O6—H1W···O1^iii^	0.86 (1)	1.94 (1)	2.801 (3)	174 (4)
O6—H2W···O5^iv^	0.86 (1)	1.91 (2)	2.741 (4)	162 (5)

Symmetry codes: (i) *x*−1, *y*, *z*−1; (ii) *x*+1, *y*, *z*; (iii) *x*+1, *y*+1, *z*; (iv) *x*, *y*−1, *z*+1.
